# Persistence of Mental Fatigue on Motor Control

**DOI:** 10.3389/fpsyg.2020.588253

**Published:** 2021-01-08

**Authors:** Thomas Jacquet, Bénédicte Poulin-Charronnat, Patrick Bard, Romuald Lepers

**Affiliations:** ^1^LEAD – CNRS UMR5022, Université Bourgogne Franche-Comté, Dijon, France; ^2^INSERM UMR 1093 CAPS, Université Bourgogne Franche-Comté, Dijon, France

**Keywords:** cognitive fatigue, recovery effect, electroencephalography (EEG), brain oscillations, event-related potentials, Fitts’ law, arm-pointing task

## Abstract

The effects of mental fatigue on both cognitive and physical performance are well described in the literature, but the recovery aspects of mental fatigue have been less investigated. The present study aimed to fill this gap by examining the persistence of mental fatigue on behavior and electrophysiological mechanisms. Fifteen participants performed an arm-pointing task consisting of reaching a target as fast as possible, before carrying out a 32-min cognitively demanding task [Time Load Dual Back (TLDB) task], and immediately, 10 and 20 min after completion of the TLDB task. During the experiment, electroencephalography was continuously recorded. The significant increase in mental fatigue feeling after the TLDB task was followed by a decrease during the 20 min of recovery without returning to premeasurement values. Brain oscillations recorded at rest during the recovery period showed an increase in both theta and alpha power over time, suggesting a persistence of mental fatigue. Arm-pointing movement duration increased gradually over time during the recovery period, indicating that behavioral performance remained impaired 20 min after the end of the cognitively demanding task. To conclude, subjective measurements indicated a partial recovery of mental fatigue following a cognitively demanding task, whereas electrophysiological and behavioral markers suggested that the effects of mental fatigue persisted for at least 20 min. While the subjective evaluation of mental fatigue is a very practical way to attest the presence of mental fatigue, electrophysiological and behavioral measures seem more relevant to evaluate the time course of mental fatigue effects.

## Introduction

Mental fatigue, which is defined as a psychobiological state caused by prolonged and/or intense periods of demanding cognitive activity and characterized by subjective feelings of “tiredness” and “lack of energy” (Boksem and Tops, 2008; Rozand and Lepers, 2016), is a common problem in everyday life. It can affect patients with cancer, Alzheimer’s or Parkinson’s disease (Chaudhuri and Behan, 2000), as well as healthy individuals, in whom it can lead to a decrease in productivity, an increase in road accidents (Dinges, 1995), or even be involved in burnout or depression (Lavidor et al., 2002).

Mental fatigue is a very complex phenomenon, and its neural mechanisms are still poorly known. They likely involve changes in brain activity involving the anterior cingulate cortex (ACC), a brain area at the interface between cognition, emotion, and motor control (Müller and Apps, 2019). Several different theories have been proposed to explain the mental fatigue-induced performance decrements; these include underload theories (Manly et al., 1999), resource theories (Sanders, 1997), motivational control theories (Kurzban et al., 2013), and dual regulation system (Ishii et al., 2014). The present study falls within the framework of resource theories, and is close to ego-depletion theory (Baumeister et al., 1998; Hagger et al., 2010). In this background, performing a cognitively fatiguing task reduces, or even under certain circumstances, depletes cerebral and cognitive resources that cannot be replenished immediately and that cannot be fully available to perform a following task (cognitive or physical). This could result in performance decrement (although some compensatory mechanisms might maintain performance, Hockey, 2013; Wang et al., 2016) and cerebral brain changes.

Since the middle of the 19th century (Davy, 1845), researchers have investigated mental fatigue in both laboratory and field conditions to better understand its effects and mechanisms. Under laboratory conditions, mental fatigue is generally induced by specific cognitive tasks. For example, the Stroop task (e.g., Rozand et al., 2015; Van Cutsem et al., 2017a), the AX-CPT (AX-continuous performance test; e.g., Marcora et al., 2009; Pageaux et al., 2013) or computerized decision-making tasks (Otani et al., 2017) have been used, generally for a duration ranging from 45–90 min, to induce mental fatigue. However, during prolonged cognitive tasks, both mental fatigue and boredom interfere, making it difficult to study mental fatigue *per se*. To differentiate between mental fatigue and boredom, shorter but more demanding cognitive tasks have been used to induce mental fatigue without boredom. For example, Borragan et al. (2016) used a cognitive task called the Time Load Dual Back (TLDB) task combining a traditional N-back working-memory updating task and an interfering second task (odd/even decision task) lasting 16 min. After completion of the TLDB task, the participants reported a significant increase in the feeling of mental fatigue, and their vigilance decreased during a subsequent psychomotor vigilance task (PVT; Borragan et al., 2017). While subjective measures [e.g., visual analog scale (VAS)] can be used to evaluate mental fatigue (Smith et al., 2019), measures based on electrophysiological recordings can provide more objective evidence of mental fatigue.

Studies using electroencephalography (EEG) have shed light on the neural mechanisms involved in mental fatigue, notably changes in brain oscillations. Mainly an increase in alpha power has consistently been observed, which might reflect a decrease in arousal and alertness (Paus et al., 1997; Boksem et al., 2005; Zhao et al., 2012). Although alpha power changes are a robust marker of mental fatigue, a recent meta-analysis suggested that an increase in theta power would be a more reliable biomarker of the presence of mental fatigue (Tran et al., 2020). According to this meta-analysis, mental fatigue results in large increases in theta power through the whole brain (i.e., frontal, central, and posterior regions), while increases in alpha power are mainly observed in central and posterior regions and to a lesser extent in frontal regions. In addition to brain oscillations, event-related potentials (ERPs) have also been considered. Consistent effects of mental fatigue have been observed on N100, N2, and P300 components. For these three components, a decrease in amplitude over time has been observed and interpreted as reflecting a top–down modulation of sensory processing for the N100 (Boksem et al., 2005; Faber et al., 2012), a decrease in cognitive control for the N2 (Boksem et al., 2006; Möckel et al., 2015) and a decrease in attention for the P300 (Murata et al., 2005; Schmidt et al., 2009). In parallel with electrophysiological changes, performance can also be impaired by mental fatigue.

It has been established that mental fatigue may negatively impact subsequent cognitive (e.g., van der Linden et al., 2003; Boksem et al., 2005) or physical (for review: Van Cutsem et al., 2017b; Pageaux and Lepers, 2018) activities. Concerning physical performance, not all physical activities are negatively impacted by mental fatigue. While previous studies have shown that mental fatigue impaired endurance performance (e.g., Marcora et al., 2009; Pageaux et al., 2014) as well as decision making (e.g., Smith et al., 2016; Harris and Bray, 2019) and motor skills (e.g., Rozand et al., 2015; Le Mansec et al., 2018), the maximal voluntary force/power production capacity seems to be preserved (e.g., Pageaux et al., 2013; Duncan et al., 2015). To evaluate the effects of mental fatigue on motor skills, Rozand et al. (2015) used an arm-pointing task consisting of reaching visual targets as fast as possible. Following mental fatigue, induced by 90 min of a modified Stroop task, these authors observed an ∼10% increase in actual movement duration, indicating an impairment of motor skills following a prolonged cognitively demanding task.

While the effects of mental fatigue on motor performance have been clearly demonstrated, questions about the persistence of these effects and the time course of recovery from mental fatigue have been poorly investigated. To our knowledge, Rivers (1896) was one of the first to address this question by evaluating the effect of a 30- or 60-min rest period following 30 min of mental work (i.e., addition calculation). Results indicated that 30 min of total rest was insufficient to neutralize the effects of mental fatigue and that 60 min of total rest only partially eliminated the effects. More recently, Smith et al. (2019) investigated the effects of three different cognitive tasks (Stroop task, AX-CPT, and PVT), lasting 60 min, on reaction times, feeling of fatigue and electrophysiological markers, as well as their persistence over time. They observed that the feeling of mental fatigue (reported on a VAS) increased immediately after the three cognitive tasks but led to a decrease in vigilance only after the Stroop task and the AX-CPT task. The level of mental fatigue decreased gradually over time, suggesting that a recovery mechanism came into play. However, the feeling of mental fatigue remained high for 10 min after the PVT, 50 min after the Stroop task, and 60 min after the AX-CPT. Concerning performance, no negative effect of mental fatigue was reported on vigilance 30 min after completion of the three different cognitively demanding tasks. These observations suggest that performance may recover faster than the subjective feeling of mental fatigue.

The main objective of the present study was to investigate the time course of mental fatigue following a 32-min cognitively demanding task on both subjective (VAS) and electrophysiological (i.e., brain oscillations) markers, as well as on motor performance. An arm-pointing task was used to evaluate the impact of mental fatigue on motor performance, due to its involvement in many activities of daily living. We firstly hypothesized that during the completion of the TLDB task, an increase over time in both theta and alpha power should be observed. It should concern all the brain regions for theta power, and more specifically central and parietal regions and to a lesser extend the frontal regions for alpha power. Concerning ERPs, we hypothesized that the amplitude of the N100, the N200, and the P300 should decrease over time as observed in previous studies (Boksem et al., 2005, 2006; Murata et al., 2005; Faber et al., 2012). Secondly, we also expected that immediately after the cognitively demanding task the subjective feeling of mental fatigue would increase and the motor performance decrease. These alterations would be associated with an increase in both theta and alpha power during the rest period immediately following the cognitively demanding task. Finally, we assumed that, during the recovery period following the completion of the cognitively demanding task, a progressive decrease in the subjective feeling of mental fatigue would be associated with a return toward initial levels of brain oscillations recorded during rest period and motor performance.

## Materials and Methods

### Participants

The study was conducted with 15 healthy active adults, eight males and seven females (mean ± SD; age: 21.9 ± 1.8 years). It included two sessions: a familiarization session and an experimental session. All participants reported normal or corrected-to-normal vision and none of them had a history of neurological disorders. They completed the Edinburgh questionnaire, which confirmed that all participants were right-handed. All participants were given instructions to sleep for at least 7 h, not to consume alcohol, and to refrain from vigorous physical activity the day before each visit. Participants were also instructed not to consume caffeine and nicotine at least 3 h before testing and were asked to declare if they had taken any medication or had any acute illness, injury, or infection. All the participants provided their written informed consent. The experiment was conducted in accordance with the Declaration of Helsinki (1964).

### The Cognitively Fatiguing Task: The Time Load Dual Back Task

The TLDB task is a dual task combining a classic N-back working-memory updating task (Kirchner, 1958) and an interfering second task (odd/even decision task). Stimuli were letters and digits displayed alternately on the screen (i.e., letter/digit/letter/…). When letters were presented, participants were instructed to press the space bar with their left hand every time the displayed letter was the same as the previous one (1-back task). When digits were displayed, participants had to press a key on the numeric keypad, using their right index and middle fingers: “1” if it was an odd number, “2” if it was even. A total of eight letters (A, C, T, L, N, E, U, and P) and eight numbers (1, 2, 3, 4, 6, 7, 8, and 9) were used. Stimulus presentation was managed by the Experiment Builder software (SR Research). Stimuli were presented in Arial font size 120, in the center of a 16-inch computer screen (refresh rate 60 Hz). They were presented in blocks of 60 trials with a break of 30 s between each block.

### The Physical Task: The Arm-Pointing Task

**FIGURE 1:**
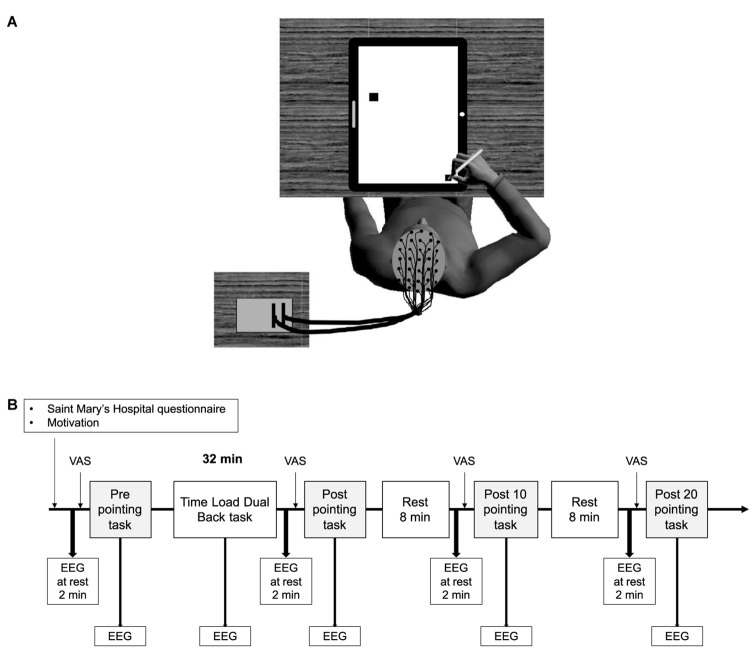
A schematic representation of the participants’ position while performing the arm-pointing task **(A)**, and an overview of the experimental protocol **(B)**. VAS, fatigue visual analog scale; EEG, electroencephalography.

Participants had to point at a target as accurately and as fast as possible with a pencil held in their right hand. They were sitting down, and in the start position their arm was aligned with their right shoulder and all targets to be pointed at were located on a 45° diagonal to the left to limit joint stress. The targets were black squares displayed on a touch screen in front of the participant (Figure 1A). The targets had different indices of difficulty (Nitschke et al., 2006), which were calculated using the formula $I\,D=\log_{2}\left(\frac{2\,D}{\text{W}}\right)$, where D represents the center-to-center distance between the start point and the target, and W represents the width of the target. Using different distances (between 5 and 32 cm) and widths (between 0.5 and 2.5 cm), 40 targets were created with eight IDs ranging from 2.5 to 6 in steps of 0.5. Each ID included five different targets with different widths and distances from the start point.

The time measured for the pointing movement began when participants took the pen from the start point and stopped when the pen touched the touch screen. If the participant landed on the target, the trial was considered as “correct”; if not, it was a miss. At each trial, the participant had to return to the start point. The pointing task lasted approximately 1.5 min.

The arm-pointing task was performed before the TLDB task, immediately after the TLDB task (Post), 10 min (Post 10) and 20 min (Post 20) after the TLDB task. For each arm-pointing task, the difficulty did not increase over time. The same trials were presented in pretest and in all the posttests, but with a different random order.

### Psychological Measurement

The Saint Mary’s Hospital Sleep Questionnaire was used to evaluate the participants’ sleep quality the night before the experiment (at each session). It contains 14 items concerning sleep quality, such as depth, awakening in the middle of the night, satisfaction, refreshed feeling upon awakening, difficulty in falling asleep, and early awakening. Participants reported that they slept on average 7 h and 41 min (±12 min).

Motivation related to the entire protocol was measured at the beginning of the experiment, using the motivation questionnaire developed and validated by Matthews et al. (2001). It has two subscales, evaluating success motivation and intrinsic motivation. Each subscale has seven questions, with a choice of five answers: 0 = not at all, 1 = a little bit, 2 = somewhat, 3 = very much, 4 = extremely. Therefore, total scores for these motivation scales range between 0 and 28. A low score reveals low motivation, and a high score indicates high motivation. Participants’ scores were 17.7 (±1.3) for success motivation and 23.3 (±0.9) for intrinsic motivation.

The participants also rated their level of mental fatigue on a VAS at four points of the experimental session: at the start of the session, after the TLDB task, and before Post 10 and Post 20 tests. The VAS was a line 100 mm long, with bipolar end anchors (0 mm = “Not mentally fatigued at all”; 100 mm = “Extremely mentally fatigued”) on which participants placed a mark to estimate their level of mental fatigue.

### Procedure

The experiment included two sessions: a familiarization session and an experimental session (Figure 1B). During the familiarization session, participants were shown the questionnaires used in the experiment, and were trained on both arm-pointing and TLDB tasks. The threshold of stimulation duration, corresponding to the highest level of TLDB task performance, was determined for each participant using the same procedure as previous studies (Borragan et al., 2016, 2017). All participants started the familiarization session with a 1500 ms presentation of each event (i.e., letters and digits) without inter-stimulus intervals. At the end of each block, if the performance accuracy was equal to or greater than 85%, the duration of the presentation was reduced by 100 ms, and so on until the accuracy was lower than 85%. At this point, participants performed two more blocks with the same duration to pursue familiarization; the threshold for the experimental session was fixed at the last successful duration. The familiarization session also aimed to reduce learning effects, which could counteract the effect of mental fatigue on performance.

The experimental session took place 48–96 h after the familiarization session, at the same time of day. Before installing the EEG recording system, participants were reminded how to perform the arm-pointing task and carried out another practice trial. While the EEG was being set up, participants completed the Saint Mary’s Hospital questionnaire and the motivation questionnaire. They were then installed on a comfortable chair in an acoustically and electrically isolated booth (Figure 1A). First, EEG activity was recorded at rest for 2 min while the participants were sat in front of a black screen, and were asked to rest and not to think about anything. After that, they were asked to rate their level of mental fatigue on the VAS and to perform the first arm-pointing task. They then performed the TLDB task, which lasted 32 min^1^ (excluding the rest periods between each block). Immediately after the TLDB task, the EEG activity was recorded at rest for 2 min, and participants rated their level of mental fatigue and performed the arm-pointing task again (Post). After 8 min of rest, the same procedure was repeated (i.e., recording EEG activity at rest for 2 min, rating mental fatigue level and arm-pointing task; Post 10). Finally, after a second rest period of 8 min, the same procedure was repeated for the last time (Post 20; see Figure 1B). During rest periods, the experimenter talked with the participant. Talking to the participant during the rest period was chosen as an ecological condition, mirroring what happens in daily life at work, for instance. The conversation was similar for each participant, and dealt with their job (or education), their hobbies (e.g., sport, nature), and their perspectives for the future (e.g., studies, jobs). These topics were always addressed in the same order.

### EEG Recording and Preprocessing

The electroencephalogram was recorded continuously through the Active Two BioSemi system from 64 electrodes in accordance with the 10–20 International system. Horizontal eye movements were monitored with electrodes placed on the outer left and right canthi, while eye blinks were monitored with an electrode placed under the left eye. Two additional electrodes were placed on the left and right mastoids (A1, A2). During recording, the BioSemi system’s common-mode sense electrode served as the reference electrode. Electrophysiological signals were digitized at 2048 Hz sampling rate and acquired with ActiView software. Offline data analyses were performed using MATLAB (MathWorks, Natick, MA, United States) and the EEGLAB toolbox (Delorme and Makeig, 2004). Continuous data were downsampled to 256 Hz, band-pass filtered at 0.01–100 Hz, and re-referenced to the average of A1 and A2. Noisy electrodes were identified with the *probability* and *spectrum* methods proposed in EEGLAB (threshold, Z = 5) and interpolated when necessary with a spherical method. To correct eye-movement artifacts, EEGLAB’s Runica routine was used to perform independent component analyses, and components reflecting eye artifacts were removed by visual inspection. Continuous data were segmented into 500 ms epochs from 50 ms before to 450 ms after the onset of the stimuli and were baseline corrected using the pre-trial period from −25 ms to 0 ms.

### Data and Analysis

#### Pointing Movement

Movement durations less than 100 ms and more than 1400 ms were excluded from the analysis. We estimated that movement duration below 100 ms and above 1400 ms were not coherent for the performed movements. Altogether, only two trials over 2400 were excluded from the data. Movement durations beyond two standard deviations (SDs) of the mean were also excluded. Analyses were performed on 88% of trials.

#### EEG Data

For the TLDB task, only trials with correct responses (93% of trials) were considered for ERP analysis. Epochs were averaged separately for each condition and each participant. ERPs were obtained by computing the mean amplitude in the time window for each ERP component, and by grand-averaging data across participants. Because no studies have previously analyzed ERPs during the TLDB task and because ERPs are stimulus and task dependent, we determined our time windows for the ERP analyses based on visual inspection, according to the peak of the ERP and its shape, to be in complete adequation with the observed ERPs. Different time windows were used for ERP components for letters on fronto-central region (N100: 90–150 ms, N200: 230–315 ms, P300: 325–445 ms) and on parietal region (N200: 130–200 ms, P300: 325–445 ms). Using the same method, ERP components were also identified for digits on fronto-central region (P50: 25–75 ms) and parietal region (N200: 135–210 ms).

For EEG spectral analysis, during the TLDB task, the 2-min rest and the arm-pointing task, EEG power of individual epochs was computed by Fast Fourier Transform (FFT), using the *spectopo* function of the EEGLAB software. It was divided into five frequency bands: delta, 1–4 Hz; theta, 4–7 Hz; alpha, 8–12 Hz; beta 13–30 Hz; and gamma, 30–40 Hz to be analyzed. Spectral analyses during the TLDB task and the arm-pointing task were performed irrespective of the occurrence of stimuli. Nine regions of interest (ROIs) were constituted to perform analysis on both ERPs and spectral data: Frontal Left (FL; mean of FP1, AF3, AF7, F3, F5, F7, FC3, FC5, FT7), Frontal Median (FM; mean of FPz, AFz, F1, Fz, F2, FC1, FCz, FC2), Frontal Right (FR: mean of FP2, AF4, AF8, F4, F6, F8, FC4, FC6, FT8), Central Left (CL: mean of C3, C5, T7, CP3, CP5, TP7), Central Median (CM: mean of C1, Cz, C2, CP1, CPz, CP2), Central Right (CR: mean of C4, C6, T8, CP4, CP6, TP8), Posterior Left (PL: mean of P9, P7, P5, P3, PO7, PO3, O1), Posterior Median (PM: mean of P1, Pz, P2, POz, Oz), and Posterior Right (PR: mean of P4, P6, P8, PO4, PO8, O2). Statistical evaluation was performed with repeated-measures ANOVAs on each ROI.

### Statistics

All data are presented as means ± standard error of the mean. Degrees of freedom were corrected using the Greenhouse–Geisser procedure when sphericity was violated (corrected degree of freedom and *p*-values are reported). Only significant effects are reported, except when non-significance is relevant for the hypotheses being tested.

To evaluate mental fatigue effects on the VAS, one-way repeated measures ANOVA with time (Pre, Post, Post 10, and Post 20) as within-subject factor was conducted. The effects of mental fatigue on movement duration and errors on arm-pointing movements were evaluated using two-way repeated measures 4 × 8 ANOVAs with time (pre, post, post 10, and post 20) and ID (2.5, 3, 3.5, 4, 4.5, 5, 5.5, and 6) as within-subject factors.

For the TLDB task, analyses were performed separately for letters and digits. Two-way repeated measures 2 × 4 ANOVAs including the within-subject factors of Stimulus type (for letters: same/different; for digits: even/odd) and time-on-task (part 1, 0–8 min; part 2, 8–16 min; part 3, 16–24 min; and part 4, 24–32 min) were performed for RTs, accuracy, and ERPs. Only ERPs interesting for mental fatigue effects are reported in the “Results” section.

Brain oscillations were analyzed using one-way repeated measures ANOVAs with time-on-task (part 1, 0–8 min; part 2, 8–16 min; part 3, 16–24 min; and part 4, 24–32 min) as within-subject factor during the TLDB task and, with time (Pre, Post, Post 10, and Post 20) for the recordings at rest. Only results about theta and alpha power are reported in the “Results” section, the other brain oscillations are reported in Supplementary Data.

All analyses were performed using the Statistical Package for the Social Sciences, version 24 for Windows (SPSS Inc., Chicago, IL, United States). Significant main effects of time and significant interactions were followed up by contrast tests, and planned comparisons using *t*-tests with Bonferroni correction for multiple comparisons as appropriate. Only significant results are reported, with adjusted *p*-values. For each ANOVA, partial eta squared is reported. Thresholds for small, moderate, and large effects were set at 0.01, 0.07, and 0.14, respectively (Cohen, 1988). Cohen’s *d* was calculated for each paired *t*-test using JASP (Version 0.9.1.0) [Windows software]. Thresholds for small, moderate, and large effects were set at 0.2, 0.5, and 0.8, respectively (Cohen, 1988).

## Results

### Mental Fatigue Assessment

#### Subjective Measure of Mental Fatigue

##### Visual analog scale

**FIGURE 2:**
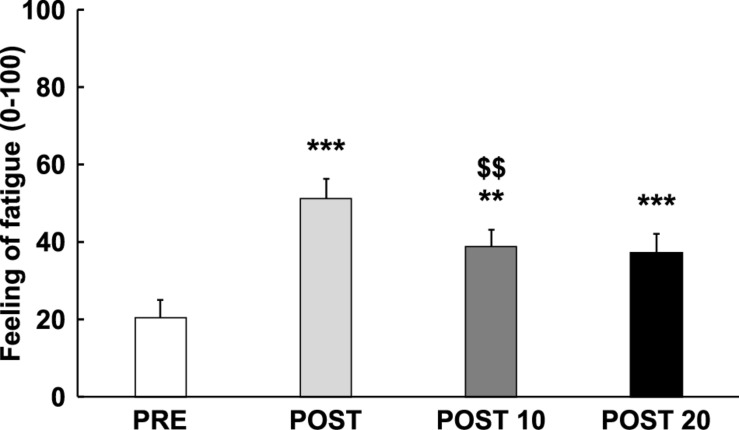
Time course of subjective feeling of mental fatigue. ** and ***: Significantly different from Pre (*p* < 0.01 and *p* < 0.001, respectively). $$: Significantly different from the previous measurement (*p* < 0.01). Data are presented as mean ± SE.

As displayed in Figure 2, analysis revealed an increase in the feeling of fatigue over time [*F*_(__3,__42__)_ = 26.784, *p* < 0.001, $\eta_{p}^{2}$ = 0.657], qualified by a cubic trend [*F*_*(1,14)*_ = 40.109, *p* < 0.001, $\eta_{p}^{2}$ = 0.741], with a significant increase in feeling-of-fatigue score between Pre and Post [*t*_(__14__)_ = 6.629, *p* < 0.001, *d* = −1.711], followed by a reduction of the fatigue score between Post and Post 10 [*t*_(__14__)_ = 4.601, *p* = 0.002, *d* = 1.188], but not between Post 10 and Post 20 [*t*_(__14__)_ = 3.876, *p* > 0.05, *d* = 0.163]. The subjective feeling of fatigue was still significantly higher at Post 10 [*t*_(__14__)_ = 4.713, *p* = 0.002, *d* = 1.217] and post 20 [*t*_(__14__)_ = 5.863, *p* < 0.001, *d* = 1.514] compared to Pre.

#### Performance During the TLDB Task

##### Reaction time

Mean reaction time was 535.2 ms (±10.0) for digits and 498.8 ms (±11.0) for letters. No significant effect of time or interaction with the factor time was observed on reaction time during the TLDB task for letters or digits (all *ps* > 0.15, $\eta_{p}^{2}$ < 0.13).

##### Accuracy

Mean accuracy was 90.9% (±0.5) and 92.8% (±0.8) for digits and letters. No significant effect of time or interaction with the factor time was observed on reaction time during the TLDB task for letters or digits (*ps* > 0.35, $\eta_{p}^{2}$ < 0.07), suggesting that performance was maintained over time.

#### EEG Data

##### Event-related potentials

###### N100

**FIGURE 3:**
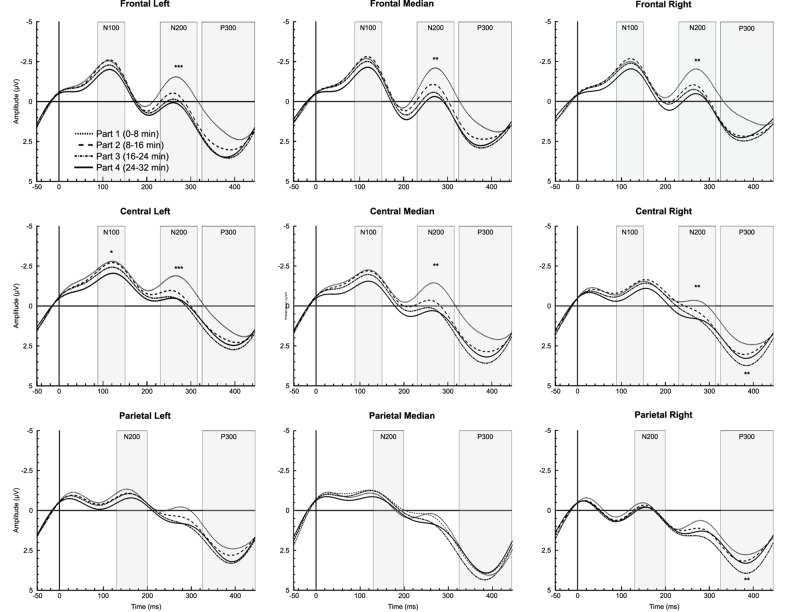
Time-on-task effect for letter stimuli during the 32-min time load dual back task in all regions of interest. *, ** and ***: Significantly time-on-task effect (*p* < 0.05, *p* < 0.01 and *p* < 0.001, respectively).

Analysis showed a significant time-on-task effect only for letters in CL region [*F*_*(3,42)*_ = 4.019, *p* = 0.033, $\eta_{p}^{2}$ = 0.223], qualified by a linear trend [*F*_*(1,14)*_ = 5.293, *p* = 0.030, $\eta_{p}^{2}$ = 0.293], reflecting a decrease in the N100 amplitude over time (Figure 3).

###### N200

**FIGURE 4:**
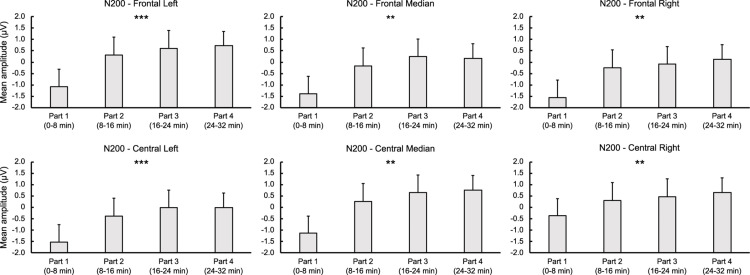
Time-on-task effect on N200 for letter stimuli during the 32-min time load dual back task in the frontal and central regions. ** and ***: Significantly time-on-task effect (*p* < 0.01 and *p* < 0.001, respectively).

For letters, ANOVA revealed a significant time-on-task effect for FL [*F*_(__3,__42__)_ = 6.755, *p* < 0.001, $\eta_{p}^{2}$ = 0.326], FM [*F*_(__3,__42__)_ = 6.388, *p* = 0.001, $\eta_{p}^{2}$ = 0.313], FR [*F*_(__3,__42__)_ = 5.755, *p* = 0.002, $\eta_{p}^{2}$ = 0.291], CL [*F*_(__3,__42__)_ = 7.064, *p* < 0.001, $\eta_{p}^{2}$ = 0.335], CM [*F*_(__1.__906,__26.__690__)_ = 6.623, *p* = 0.005, $\eta_{p}^{2}$ = 0.321], and CR regions [*F*_(__3,__42__)_ = 6.274, *p* = 0.001, $\eta_{p}^{2}$ = 0.309], indicating a linear decrease in N200 amplitude over time for all these regions (*p*s < 0.012,$\eta_{p}^{2}$ > 0.378). For digits, analysis showed a significant main effect of time-on-task for LF [*F*_(__3,__42__)_ = 4.278, *p* = 0.021, $\eta_{p}^{2}$ = 0.234] and CL regions [*F*_(__3,__42__)_ = 4.090, *p* = 0.020, $\eta_{p}^{2}$ = 0.226], qualified by a linear trend for both FL [*F*_(__1,__14__)_ = 7.576, *p* = 0.016, $\eta_{p}^{2}$ = 0.351] and CL regions [*F*_(__1,__14__)_ = 7.882, *p* = 0.019, $\eta_{p}^{2}$ = 0.360] indicating, as for letters, a decrease in the N200 amplitude over time (see Figures 3, 4).

###### P300

A significant time-on-task effect was reported only for letters on the P300 amplitude for CR [*F*_(__3,__42__)_ = 8.465, *p* = 0.002, $\eta_{p}^{2}$ = 0.377], qualified by a linear trend [*F*_(__1,__14__)_ = 11.415, *p* = 0.005, $\eta_{p}^{2}$ = 0.449], indicating an increase in the P300 amplitude over time. On PR region, analysis indicated a significant time-on-task effect on the P300 amplitude [*F*_(__3,__42__)_ = 5.854, *p* = 0.004, $\eta_{p}^{2}$ = 0.295], qualified by a quadratic trend [*F*_(__1,__14__)_ = 9.208, *p* = 0.009, $\eta_{p}^{2}$ = 0.397], reflecting an increase in P300 amplitude over time between the first and the second part [*t*(_14__)_ = −2.460, *p* = 0.028, *d* = −0.635], and between the second and the third part of the task [*t*_(__14__)_ = −5.863, *p* = 0.022, *d* = −0.667], but a decrease in the P300 amplitude between the third and the fourth part [*t*_(__14__)_ = 2.164, *p* = 0.048, *d* = −0.559] (Figure 3).

##### Brain oscillations

All the results of brain oscillations are presented in the Supplementary Table S1. Only significant differences related to brain oscillations useful to test our hypothesis, i.e., theta and alpha powers, are presented below.

###### Theta

**FIGURE 5:**
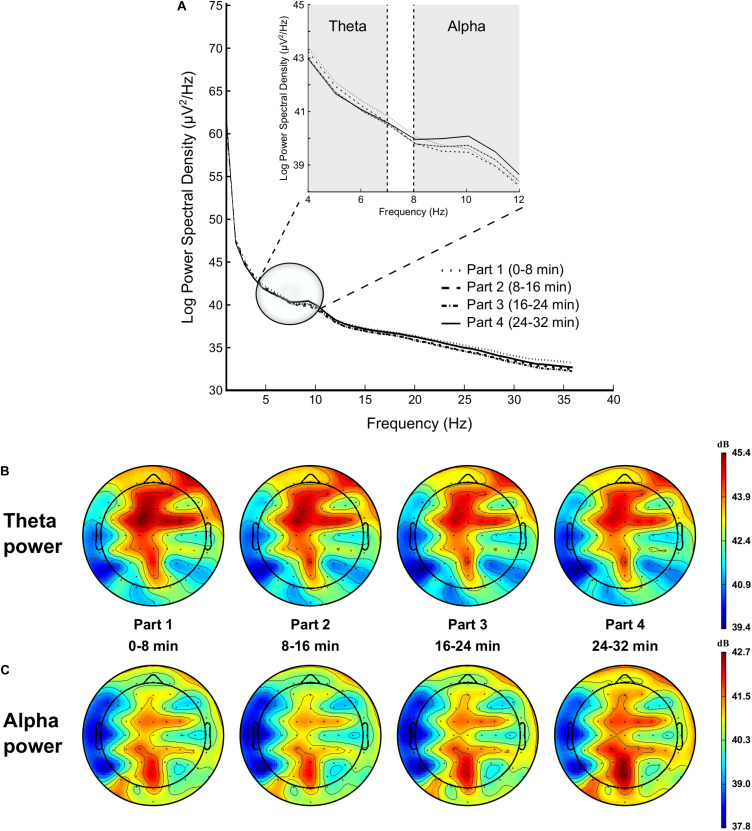
Modulation of brain power spectrum for part 1 (0–8 min), part 2 (8–16 min), part 3 (16–24 min), and part 4 (24–32 min) of the time load dual back task **(A)**, with specifically time course of the theta power **(B)** and alpha power **(C)**.

Analysis showed a significant time-on-task effect on FL [*F*_(__3,__42__)_ = 6.779, *p* < 0.001, $\eta_{p}^{2}$ = 0.326], FM [*F*_(__3,__42__)_ = 12.828, *p* < 0.001, $\eta_{p}^{2}$ = 0.478], FR [*F*_(__3,__42__)_ = 11.840, *p* < 0.001, $\eta_{p}^{2}$ = 0.458], CL [*F*_(__3,__42__)_ = 13.411, *p* = 0.028, $\eta_{p}^{2}$ = 0.489], CM [*F*_(__3,__42__)_ = 13.570, *p* < 0.001, $\eta_{p}^{2}$ = 0.492], CR [*F*_(__3,__42__)_ = 9.616, *p* < 0.001, $\eta_{p}^{2}$ = 0.407], PM [*F*_(__3,__42__)_ = 7.754, *p* < 0.001, $\eta_{p}^{2}$ = 0.356], and PR regions [*F*_(__1.__888,__26.__438__)_ = 5.908, *p* = 0.008, $\eta_{p}^{2}$ = 0.297], qualified for all these regions by a linear trend (all *ps* < 0.011, $\eta_{p}^{2}$ > 0.230), indicating a decrease in theta power over time (see Figure 5).

###### Alpha

ANOVA revealed a significant time-on-task effect on FL [*F*_(__1.__699,__23.__781__)_ = 6.827, *p* = 0.006, $\eta_{p}^{2}$ = 0.328], FM [*F*_(__1.__447,__20.__253__)_ = 7.351, *p* = 0.007, $\eta_{p}^{2}$ = 0.344], FR [*F*_(__1.__600,__22.__397__)_ = 5.811, *p* = 0.013, $\eta_{p}^{2}$ = 0.293], CL [*F*_(__1.__447,__20.__256__)_ = 4.849, *p* = 0.028, $\eta_{p}^{2}$ = 0.257], CM [*F*_(__1.__601,__22.__426__)_ = 6.528, *p* = 0.008, $\eta_{p}^{2}$ = 0.318], CR [*F*_(__1.__617,__22.__634__)_ = 5.426, *p* = 0.016, $\eta_{p}^{2}$ = 0.279], PL [*F*_(__1.__579,__22.__108__)_ = 5.329, *p* = 0.018, $\eta_{p}^{2}$ = 0.276], and PM regions [*F*_(__1.__621,__22.__692__)_ = 7.779, *p* = 0.004, $\eta_{p}^{2}$ = 0.357]. This main effect of time-on-task was qualified by a quadratic trend for all these regions (all *ps* < 0.020, $\eta_{p}^{2}$ > 0.330), highlighting a slight decrease in alpha power between the first and the second part of the TLDB task, followed by an increase in alpha power over time (see Figure 5).

### Effect of Mental Fatigue on the Arm-Pointing Task

#### Movement Duration

**FIGURE 6:**
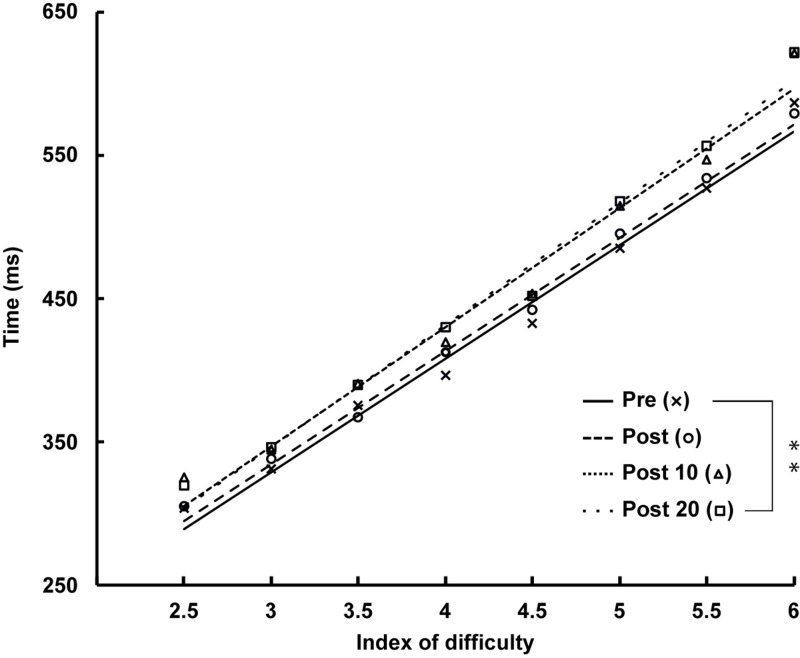
Time course of the duration of arm-pointing movement at Pre, Post, Post 10, and Post 20 with a significant effect of index of difficulty (ID) (*p* < 0.001). ^∗∗^: Significantly different from Pre (*p* < 0.01).

There was a significant main effect of ID [*F*_(__1.__245,__17.__428__)_ = 136.045, *p* < 0.001, $\eta_{p}^{2}$ = 0.907] qualified by a linear trend [*F*_(__1,__14__)_ = 10.607, *p* = 0.006, $\eta_{p}^{2}$ = 0.431], indicating an increase in movement duration with increasing ID, accompanied by a main effect of time [*F*_(__3,__42__)_ = 5.259, *p* = 0.005, $\eta_{p}^{2}$ = 0.273]. The decomposition of this time effect revealed a linear increase in the duration of arm-pointing movements over time [*F*_(__1,__14__)_ = 10.607, *p* = 0.006, $\eta_{p}^{2}$ = 0.431] as shown by comparison with Pre [*vs.* Post: *t*_(__14__)_ = −0.717, *p* = 1.000, *d* = −0.185; *vs.* Post 10: *t*_(__14__)_ = −2.527, *p* = 0.121, *d* = −0.653], up to significance at Post 20 measurement [*t*_(__14__)_ = −2.977, *p* < 0.050, *d* = −0.769] (see Figure 6).

#### Pointing Errors

There was a significant main effect of ID on errors during the pointing task [*F*_(__2.__305,__32.__275__)_ = 26.782, *p* < 0.001, $\eta_{p}^{2}$ = 0.657]. The decomposition of this main effect revealed a significant linear effect, with more errors as the ID of the target increased [*F*_(__1,__14__)_ = 48.455, *p* < 0.001, $\eta_{p}^{2}$ = 0.776]. However, analyses showed no significant effect of time or ID × time interaction.

### Effects of Mental Fatigue on Brain Oscillations at Rest

All the results of brain oscillations at rest are presented in the Supplementary Table S2. Significant differences related to alpha and theta powers are presented below.

#### Theta

**FIGURE 7:**
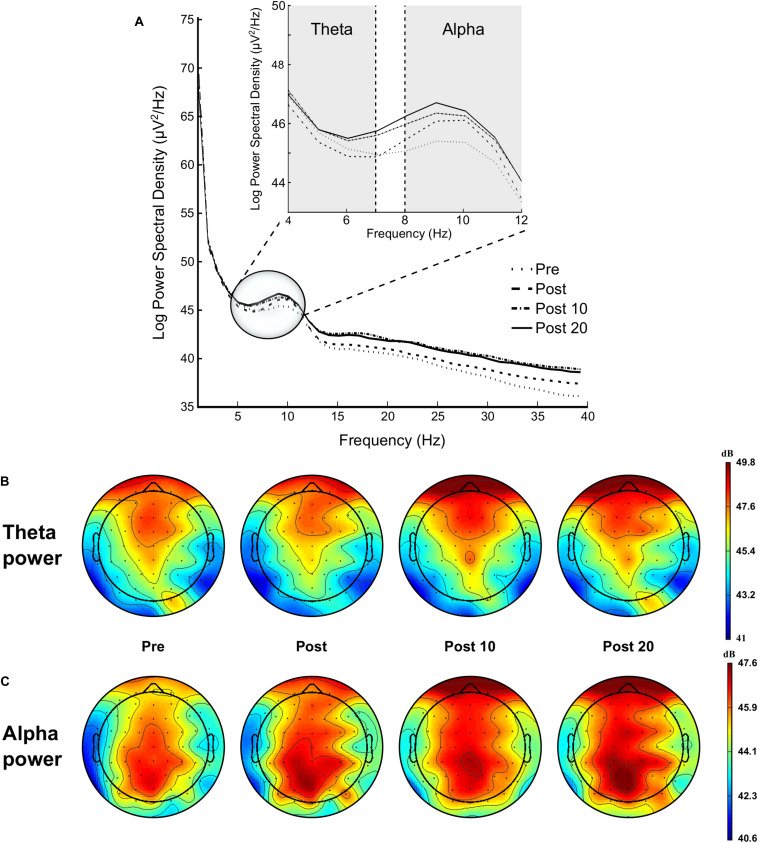
Modulation of brain power spectrum during the 2-min rest periods at Pre, Post, Post 10, and Post 20 **(A)**, with specifically time course of the theta power **(B)** and alpha power **(C)**.

Analysis showed a significant time effect on FL region [*F*_*(3,42)*_ = 4.359, *p* = 0.009, $\eta_{p}^{2}$ = 0.237], qualified by a linear trend [*F*_*(1,14)*_ = 7.220, *p* = 0.018, $\eta_{p}^{2}$ = 0.340], indicating an increase in theta power over time (see Figure 7).

#### Alpha

ANOVA revealed a significant time effect on FL [*F*_*(3,42)*_ = 4.641, *p* = 0.005, $\eta_{p}^{2}$ = 0.261], FM [*F*_*(3,42)*_ = 3.174, *p* = 0.034, $\eta_{p}^{2}$ = 0.185], FR [*F*_*(3,42)*_ = 4.177, *p* = 0.011, $\eta_{p}^{2}$ = 0.230], CL [*F*_*(3,42)*_ = 4.017, *p* = 0.013, $\eta_{p}^{2}$ = 0.223], CR [*F*_*(3,42)*_ = 3.107, *p* = 0.036, $\eta_{p}^{2}$ = 0.182], PM [*F*_*(3,42)*_ = 3.107, *p* = 0.037, $\eta_{p}^{2}$ = 0.312], and PR regions [*F*_*(3,42)*_ = 6.711, *p* = 0.021, $\eta_{p}^{2}$ = 0.324]. This main effect of time-on-task was qualified by a linear trend for all these regions (*ps* < 0.011, $\eta_{p}^{2}$ > 0.230], indicating an increase in alpha power over time (see Figure 7).

## Discussion

The objective of the present study was to investigate the time course of mental fatigue effects following a cognitively demanding task (i.e., TLDB task) on subjective and electrophysiological markers as well as on arm-pointing task performance. The results showed that the subjective feeling of fatigue increased following the cognitively demanding task and then progressively decreased during the 20 min of recovery, but without returning to initial values. Brain oscillations during the rest periods showed a linear increase in both theta and alpha powers, suggesting that mental fatigue persisted after completion of the cognitively demanding task, and that no recovery mechanism really occurred. Contrary to expectations, motor control performance was worse during the recovery period than immediately after the cognitively demanding task, movements becoming even slower 20 min after completion of the task.

### Subjective Feeling of Mental Fatigue

In agreement with previous studies (Borragan et al., 2016, 2017), 32 min on the TLDB task induced an increase in the subjective feeling of mental fatigue, indicating that this cognitively demanding task successfully induced mental fatigue. The feeling of mental fatigue decreased 10 min after completion of the task and remained above the initial value 20 min later, indicating that recovery was not complete. Recently, similar effects were observed by Smith et al. (2019), who found that the subjective feeling of mental fatigue following 45 min performing mentally fatiguing tasks remained higher than pre-treatment values for 10 min after a PVT, 50 min after a Stroop task and 60 min after an AX-CPT task. These observations indicate that the recovery of the subjective level of mental fatigue may require more time than the duration of the cognitively demanding task itself.

### Performance During the Cognitively Demanding Task

During the 32-min TLDB task, maintenance of performance (reaction time and accuracy) was observed, in contrast to previous studies by Borragan et al. (2016, 2017), who found a decrease in accuracy over time. It is worth noting that not all studies investigating mental fatigue found impaired performance during the cognitively demanding tasks used to induce mental fatigue; while some studies reported a decrease in performance (e.g., Marcora et al., 2009; Head et al., 2017), others found that it was maintained (e.g., Pageaux et al., 2015). Wang et al. (2016) postulated that the maintenance of performance despite mental fatigue could be explained by a compensatory strategy reflected by an increase in anterior frontal brain activity during a prolonged cognitive task. Herlambang et al. (2019) found that performance of a prolonged cognitive task was maintained when participants had a high level of motivation, and decreased when their motivation was low. These observations suggest that motivation is an important factor when considering mental fatigue and its effects. In the present study, the participants showed high motivation to perform the experiment (success motivation = 17.7 ± 1.3 and intrinsic motivation = 23.3 ± 0.9 overall on a 0–28 scale), which could explain the maintenance of performance during the cognitively demanding task.

### Electrophysiological Markers During the Cognitively Demanding Task

In addition to subjective and behavioral measures, changes in ERP amplitude during the cognitively demanding task could provide an objective marker of mental fatigue, and explain the maintenance of behavioral performance over time. In the present study, the decrease in N100, considered as an indicator of mental fatigue (Boksem et al., 2005; Faber et al., 2012), might reflect an increase in mental workload. However, the maintenance of performance (i.e., reaction time and accuracy) suggests that a compensatory mechanism was involved during the cognitively demanding task. Previous studies reported a decrease in P300 amplitude (Koelega et al., 1992; Murata et al., 2005), interpreted as a decrease in attention over time during a prolonged cognitive task (Zhao et al., 2012), associated to a decline in behavioral performance with mental fatigue (Cheng et al., 2007; Guo et al., 2015). In the present study, the linear increase in P300 amplitude reported in central regions for letter stimulus could be attributed to an increase in cerebral resources needed to maintain task performance despite progressive mental fatigue.

In addition to the changes observed in the ERP components, brain oscillations also seemed to indicate a mental fatigue state. The increase in alpha power with mental fatigue induction has been widely reported in the literature and has been interpreted as reflecting a decrease in arousal and alertness (Paus et al., 1997; Lal and Craig, 2002; Boksem et al., 2005; Trejo et al., 2005; Zhao et al., 2012). Another interpretation of the increase in alpha power during the TLDB task could be that, in presence of mental fatigue, the participants had to allocate more cognitive resources to maintain their performance. However, an increase in alpha power has been also observed with performance impairment (Boksem et al., 2005; Zhao et al., 2012). The increase in alpha power was also observed when comparing rest periods (i.e., before and after the cognitively demanding task) in the present study but also in previous studies (e.g., Li et al., 2020). These findings suggest that the increase in alpha power observed in mental fatigue studies, and more especially in time-on-task design, is likely not related to the allocation of more cognitive resources to maintain task performance. This increase in alpha power with mental fatigue has often been associated with an increase in theta power mainly over the prefrontal cortex (Lal and Craig, 2002; Trejo et al., 2005). Furthermore a recent meta-analysis suggests that an increase in theta power is a robust biomarker of mental fatigue, whereas an increase in alpha power is a second-line biomarker due to considerable individual variability (Tran et al., 2020). In the present study, a decrease in theta power was reported over time in frontal regions during the cognitively demanding task. Although theta power decrease has previously been attributed to boredom (Katahira et al., 2018), the relatively short duration of the cognitively demanding task used here (i.e., 32 min) limited the boredom effect.

### Brain Oscillations at Rest

In contrast to the brain oscillations analyzed during the cognitively demanding task, those recorded at rest showed an increase in both theta and alpha powers, confirming previous results of the effects of mental fatigue on brain oscillations (Tran et al., 2020). This finding supports the importance of recording brain oscillations not only during the cognitively demanding task but also during the subsequent recovery period, in order to avoid confusion between changes due to the task *per se*, and those actually related to mental fatigue.

While the subjective feeling of mental fatigue decreased during the recovery period, the increase in both theta and alpha powers recorded at rest suggests that subjective (i.e., feeling of mental fatigue) and objective (i.e., brain oscillations) markers could provide contradictory data for interpreting the state of mental fatigue following a cognitively demanding task. Although evaluation of subjective mental fatigue using a VAS is considered to be the most practical way of assessing mental fatigue induction (Smith et al., 2019), it appears that objective electrophysiological markers of mental fatigue, such as changes in brain oscillations, could lead to a different conclusion than subjective evaluation. The present results suggest that when studying mental fatigue, it could be relevant to use different subjective and objective measures to have a more comprehensive view of the effects of mental fatigue.

### Absence of Motor Performance Recovery

In accordance with the literature, we observed an increase in the duration of movement as the index of difficulty increased during an arm-pointing task (Rozand et al., 2015; Gueugneau et al., 2017) and maintenance of the speed/accuracy trade-off despite the presence of mental fatigue (Rozand et al., 2015, 2016).

In the present study, movement duration remained constant immediately after the cognitively demanding task, which differs from the results of Rozand et al. (2015). Those authors found that after 90 min of a modified Stroop task, the duration of arm-pointing movement increased by an average of 9%, irrespective of the index of difficulty of the target. The impairment of motor control under mental fatigue was confirmed in a second study by Rozand et al. (2016), who observed a 6% increase in movement duration after a cognitively demanding task inducing mental fatigue. However, the authors indicated that the negative effects of mental fatigue on movement duration seemed to be very short-lived. Indeed, from the second arm-pointing movement, movements were as fast as pretest measurements. In our study, the effects of mental fatigue on motor control were evaluated using the average time of 40 arm-pointing movements. Thus, if a mental fatigue effect occurred only on the first movement it was not enough to be observed on the average of 40 arm-pointing movements. It is worth noting that our experimental design does not allow us to verify this hypothesis, because movement times depend on the ID, and the ID associated with the first movement changed at each pointing task due to the use of different random orders.

When the arm-pointing task was repeated 10 and 20 min after the cognitively demanding task, movement duration was more and more affected, becoming significantly slower 20 min after the task compared to initial performance. A possible interpretation could be that the deterioration of the pointing performance over time was related to muscular fatigue. However, in our pilot experiment the participants repeated five times an arm-pointing task, consisting in 40 movements, after 16 min of TLDB task without a deterioration of the performance. This observation suggested that the decrease in performance in this present study was not related to the repetition of arm-pointing movements or to a possible muscular fatigue, but rather to the effects of mental fatigue.

Neuroanatomically, one possible explanation could be the involvement of the ACC, one of the main brain structures involved in the mental fatigue process (Lorist et al., 2005; Boksem and Tops, 2008; Marcora et al., 2009). Although Tanaka et al. (2014) found reduced activation of the ACC with mental fatigue during a subsequent physical activity, Johnston et al. (2007) observed that switching tasks could lead to an increase in ACC activity. This increase could lead to more resources being engaged to perform the new task. In the present study, the cognitively demanding task was different from the arm-pointing task, and as a consequence the task switching might have increased the activity of the ACC and, in the short term, could have counteracted the effect of mental fatigue. Immediately after the cognitively demanding task, this could result in more resources engaged in arm-pointing movements and could have boosted speed of movements, even in the presence of mental fatigue. During the recovery period, there was no longer task switching due to the repetition of the arm-pointing task. This might lead to a reduced ACC activity, which no longer counteracted the effect of mental fatigue. The persistence of mental fatigue could explain the impaired motor performance 20 min after completion of the cognitively demanding task.

## Conclusion

The present study found that a 32-min cognitively demanding task induced mental fatigue. As predicted, the subjective feeling of mental fatigue decreased gradually during the 20 min recovery period but remained higher than before the cognitively demanding task. The increase in both theta and alpha power of brain oscillations during the recovery period suggests that participants remained mentally fatigued despite their lower subjective feeling of mental fatigue. The persistence of fatigue during the 20 min period following the cognitively demanding task is in accordance with behavioral results of the arm-pointing task indicating that motor control performance remained impaired. These findings indicate that even if subjective indicators of mental fatigue, such as VASs, are a practical method for assessing mental fatigue, objective indicators such as behavioral and electrophysiological markers are required to have a better characterization of the state of mental fatigue following cognitively demanding tasks.

## Data Availability Statement

The raw data supporting the conclusions of this article will be made available by the authors, without undue reservation.

## Ethics Statement

Ethical review and approval was not required for the study on human participants in accordance with the local legislation and institutional requirements. The patients/participants provided their written informed consent to participate in this study.

## Author Contributions

TJ was involved in the design of the study, data collection, analysis, and interpretation, as well as in the draft of the main document. BP-C participated to the elaboration of the experimental design, data collection, analysis, data interpretation, and in drafting the document. PB took part in study programming and data analyses. RL participated to the elaboration of experimental design, the data interpretation, and had also an active role in the manuscript writing. All the authors approved the final version and agree to be accountable for all aspects of the work.

## Conflict of Interest

The authors declare that the research was conducted in the absence of any commercial or financial relationships that could be construed as a potential conflict of interest.

## Footnotes

1 In a pilot experiment, 16 min of the TLDB task were used to induce mental fatigue as in Borragan et al. (2016). However, while 16 min of the TLDB task induced a subjective feeling of mental fatigue, it was not long enough to induce a decrement in arm-pointing task performance. The duration of the TLDB task was doubled to 32 min and, under these circumstances, both an increase in subjective mental fatigue and a decrement in arm-pointing task performance were observed.
