# Supplementary material for: Persistence of Mental Fatigue on Motor Control
Source: Front Psychol. 2021 Jan 8;11:588253. doi: 10.3389/fpsyg.2020.588253 (PMC7820710; doi:10.3389/fpsyg.2020.588253)
Supplement: Supplementary file 1 [file Table_1.pdf]

Supplementary data

Supplementary Table 1. Changes on brain oscillations during the time load dual back (TLDB) task on delta, beta, and gamma power for all regions of interest.

**Time-on-task effect on brain oscillations during the TLDB task** p (η_p^2)								
**Delta**								
Frontal Left	Frontal Median	Frontal Right	Central Left	Central Median	Central Right	Posterior Left	Posterior Median	Posterior Right
.288 (.084)	.235 (.095)	.229 (.097)	.147 (.129)	.259 (.092)	.319 (.079)	.494 (.048)	.254 (.093)	.178 (.118)
Interpretation: no significant time effect on delta power.								
**Beta**								
Frontal Left	Frontal Median	Frontal Right	Central Left	Central Median	Central Right	Posterior Left	Posterior Median	Posterior Right
.002 (.287)	.053 (.190)	.011 (.231)	< .001 (.459)	.025 (.226)	.007 (.244)	.074 (.165)	.114 (.143)	.041 (.202)
Interpretation: Decrease in beta power at the beginning of the TLDB task followed by an increase in beta power evidenced by a quadratic trend (all *ps* < .030; all η_p^2 > .30).								
**Gamma**								
Frontal Left	Frontal Median	Frontal Right	Central Left	Central Median	Central Right	Posterior Left	Posterior Median	Posterior Right
.007 (.244)	.058 (.183)	.013 (.224)	< .001 (.413)	.016 (.245)	.005 (.263)	.022 (.203)	.132 (.133)	.036 (.207)
Interpretation: Decrease in gamma power at the beginning of the TLDB task followed by an increase in gamma power evidenced by a quadratic trend (all *ps* < .045; all η_p^2 > .25).								

Supplementary Table 2. Changes on brain oscillations at rest on delta, beta, and gamma power for all regions of interest.

**Time effect on brain oscillations at rest** p (η_p^2)								
**Delta**								
Frontal Left	Frontal Median	Frontal Right	Central Left	Central Median	Central Right	Posterior Left	Posterior Median	Posterior Right
.209 (.101)	.090 (.141)	.186 (.107)	.679 (.035)	.200 (.104)	.253 (.091)	.235 (.095)	.401 (.067)	.874 (.016)
Interpretation: no significant time effect on delta power.								
**Beta**								
Frontal Left	Frontal Median	Frontal Right	Central Left	Central Median	Central Right	Posterior Left	Posterior Median	Posterior Right
.004 (.335)	.006 (.312)	002 (.298)	.004 (.337)	.029 (.237)	.001 (.321)	.001 (.319)	.034 (.224)	.009 (.238)
Interpretation: significant linear increase in beta power over time (all *ps* < .033; all η_p^2 > .286).								
**Gamma**								
Frontal Left	Frontal Median	Frontal Right	Central Left	Central Median	Central Right	Posterior Left	Posterior Median	Posterior Right
< .001 (.355)	.001 (.322)	.001 (.318)	< .001 (.355)	.002 (.303)	< .001 (.382)	< .001 (.381)	.001 (.304)	.001 (.252)
Interpretation: significant linear increase in gamma power over time (all *ps* < .015; all η_p^2 > .356).								
